# Community-based Rehabilitation Intervention for people with Schizophrenia in Ethiopia (RISE): study protocol for a cluster randomised controlled trial

**DOI:** 10.1186/s13063-016-1427-9

**Published:** 2016-06-24

**Authors:** Laura Asher, Mary De Silva, Charlotte Hanlon, Helen A. Weiss, Rahel Birhane, Dawit A. Ejigu, Girmay Medhin, Vikram Patel, Abebaw Fekadu

**Affiliations:** Centre for Global Mental Health, Department of Population Health, London School of Hygiene and Tropical Medicine, London, UK; Department of Psychiatry, School of Medicine, College of Health Sciences, Addis Ababa University, Addis Ababa, Ethiopia; Centre for Global Mental Health, Health Services and Population Research Department, Institute of Psychiatry, Psychology and Neuroscience, King’s College London, London, UK; MRC Tropical Epidemiology Group, Department of Infectious Disease Epidemiology, London School of Hygiene and Tropical Medicine, London, UK; Department of Pharmacology, St Paul’s Hospital Millennium Medical College, Addis Ababa, Ethiopia; Aklilu Lemma Institute of Pathobiology, Addis Ababa University, Addis Ababa, Ethiopia; Public Health Foundation of India, New Delhi, India; Sangath, Goa, India; Centre for Affective Disorders, Department of Psychological Medicine, Institute of Psychiatry, Psychology and Neuroscience, King’s College London, London, UK

**Keywords:** Schizophrenia, Psychosis, Community-based rehabilitation, Disability, Cluster randomised trial, Ethiopia, Low-income country

## Abstract

**Background:**

Care for most people with schizophrenia is best delivered in the community and evidence-based guidelines recommend combining both medication and a psychosocial intervention, such as community-based rehabilitation. There is emerging evidence that community-based rehabilitation for schizophrenia is effective at reducing disability in middle-income country settings, yet there is no published evidence on the effectiveness in settings with fewer mental health resources. This paper describes the protocol of a study that aims to evaluate the effectiveness of community-based rehabilitation as an adjunct to health facility-based care in rural Ethiopia.

**Methods:**

This is a cluster randomised trial set in a rural district in Ethiopia, with sub-district as the unit of randomisation. Participants will be recruited from an existing cohort of people with schizophrenia receiving treatment in primary care. Fifty-four sub-districts will be randomly allocated in a 1:1 ratio to facility-based care plus community-based rehabilitation (intervention arm) or facility-based care alone (control arm). Facility-based care consists of treatment by a nurse or health officer in primary care (antipsychotic medication, basic psychoeducation and follow-up) with referral to a psychiatric nurse-led outpatient clinic or psychiatric hospital when required. Trained community-based rehabilitation workers will deliver a manualised community-based rehabilitation intervention, with regular individual and group supervision. We aim to recruit 182 people with schizophrenia and their caregivers. Potential participants will be screened for eligibility, including enduring or disabling illness. Participants will be recruited after providing informed consent or, for participants without decision-making capacity, after the primary caregiver gives permission on behalf of the participant. The primary outcome is disability measured with the 36-item WHO Disability Assessment Schedule (WHODAS) version 2.0 at 12 months. The sample size will allow us to detect a 20 % difference in WHODAS 2.0 scores between treatment arms with 85 % power. Secondary outcomes include change in symptom severity, economic activity, physical restraint, discrimination and caregiver burden.

**Discussion:**

This is the first trial of community-based rehabilitation for schizophrenia and will determine, as a proof of concept, the added value of community-based rehabilitation compared to facility-based care alone in a low-income country with scarce mental health resources.

**Trial registration:**

Clinical Trials.gov Identifier NCT02160249. Registered on 3 June 2014.

**Electronic supplementary material:**

The online version of this article (doi:10.1186/s13063-016-1427-9) contains supplementary material, which is available to authorized users.

## Background

Schizophrenia can be a severe, chronic and disabling condition, which places a high social and economic burden on individuals [1], families [2] and society. The majority of people with schizophrenia in sub-Saharan Africa do not have access to adequate care [3]. Over half of the people with schizophrenia in Ethiopia experience continuous or episodic illness over a 10-year period [4], where the treatment gap is estimated to be 90 % [5, 6]. Mortality is high; the standardised mortality ratio for people with schizophrenia in Ethiopia is twice that of the general population [7]. People with schizophrenia are also likely to experience stigma and discrimination [8, 9] and human rights violations [10].

According to global consensus, supported by scientific review of the evidence and the experience of mental health system experts, a comprehensive mental health system includes both community- and hospital-based components of care [11, 12] and should incorporate both medication and psychosocial interventions [13, 14]. Relatively low-intensity psychosocial community-based programmes, which are likely to be most feasible, have been shown to be effective in low- and middle-income country (LMIC) settings [15–17]. To date such programmes have typically involved four groups: the patient, family members, a community-based non-specialist key worker and a psychiatrist [14]. In particular, the World Health Organisation’s (WHO’s) mental health Gap Action Programme (mhGAP) recommends community-based rehabilitation (CBR) as an adjunct to medication for schizophrenia [18]. CBR is a general approach with the aim to improve the quality of life and social inclusion of people with disabilities [19] and is typically delivered by trained lay workers from the local community. Key pillars of a CBR programme include interventions that promote health, education, livelihood, and social life. In addition there is a cross-cutting emphasis on empowerment, such as supporting people with disabilities to make their own decisions. CBR is put into practice through the joint endeavours of people with disabilities, their caregivers, community members and relevant governmental and non-governmental services, including health services [20].

CBR programmes have traditionally focussed on people with physical disabilities. There is increasing recognition that people with mental disorders may also receive benefit from a service model that integrates mental health and economic development [21, 22]. CBR may impact on clinical and disability outcomes in people with schizophrenia by improving understanding of the illness, increasing adherence to antipsychotic medication, reducing stigma and improving social functioning.

Globally there are a few examples of CBR programmes for people with mental disorders [17, 23–30]. A systematic review found that CBR may improve clinical outcomes and functioning for schizophrenia, dementia and intellectual disabilities in LMICs [20]. However, no randomised controlled trials (RCTs) of CBR for schizophrenia that involved community mobilisation (defined as ‘a strategy which aims to engage community members and empower them for change or action’ [19]), or focussed primarily on any CBR pillar other than health, were included [20]. Furthermore, there were no studies set in countries defined by the World Bank as being low-income [20]. The more recent COmmunity care for People with Schizophrenia in India (COPSI) trial [31], a study from India (a middle-income country), found that collaborative community care modestly improved disability and symptoms in people with schizophrenia [17]. The greatest effects were seen in rural areas with fewer formal mental health resources. Whilst influenced by CBR, the intervention did not include community mobilisation, and participants had access to psychiatrists as a key component of care.

To our knowledge there has been no previous randomised trial investigating the impact of a comprehensive CBR programme, including both home-based care and a structured community mobilisation element, on outcomes in people with schizophrenia. Furthermore, the effectiveness of CBR for schizophrenia has not previously been examined in a low-income setting, such as Ethiopia, which has minimal formal mental health resources. Currently, most people with schizophrenia in rural Ethiopia will never have access to a psychiatrist, psychiatric nurse or other mental health professional. However, mental health care is being scaled up in Ethiopia by training general health workers, largely in primary care, to deliver care for people with mental disorders. This process illustrates the Ethiopian Ministry of Health’s efforts to improve access to mental health care [32].

Extensive formative research using a variety of methods nested within a Theory of Change framework has enabled the design of a culturally and contextually appropriate CBR intervention for people with schizophrenia that is acceptable and feasible to service users and providers in rural Ethiopia [33, 34]. The CBR intervention, which includes home-based care, community mobilisation and family support groups, has been piloted. This paper presents the protocol for the Rehabilitation Intervention for people with Schizophrenia in Ethiopia (RISE) project, a cluster randomised trial, which will evaluate the effectiveness of this CBR intervention.

## Objectives

### Primary objective

To evaluate the effectiveness of CBR as an adjunct to facility-based care (FBC), compared to FBC alone, in reducing disability related to schizophrenia at 12 months, measured by the WHO Disability Assessment Schedule (WHODAS) version 2.0 in patients with evidence of poor response or lack of engagement in care over the preceding 6 months.

### Secondary objectives

1. To evaluate the effectiveness of CBR plus FBC compared to FBC alone in reducing clinical symptoms, reducing relapse, increasing medication adherence, improving economic activity, reducing physical restraint, reducing discrimination, and improving nutritional status in people with schizophrenia.
2. To evaluate the effectiveness of CBR plus FBC compared to FBC alone in reducing family burden, stigma and depression, and improving economic activity in caregivers of people with schizophrenia
3. To explore the acceptability and feasibility of CBR from the perspective of (1) those receiving the treatment, (2) their families, and (3) those delivering the treatment.
4. To determine the cost-effectiveness of CBR plus FBC compared to FBC alone.
5. To investigate the process through which CBR achieves its impact.

### Primary hypothesis

People with schizophrenia who receive CBR in addition to FBC will experience greater reductions in disability compared to those who receive only FBC, to the order of 20 % absolute difference in WHODAS 2.0 score between groups, over a 12-month period.

## Methods

### Study design

**Fig. 1:**
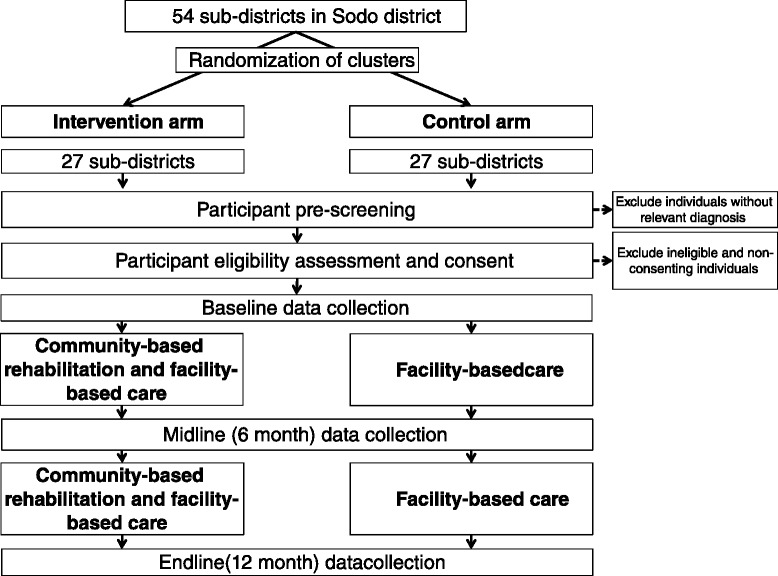
RISE flow chart

The design is a cluster randomised controlled trial with sub-district (pre-defined administrative unit consisting of several villages together) as the unit of randomisation. The study flow chart is presented in Fig. 1. From 58 sub-districts in the study district, four sub-districts were sites for the pilot and the remaining 54 sub-districts will be included for the actual trial; 27 will be randomly allocated to the intervention arm (FBC plus CBR) and 27 randomly allocated to the control arm (FBC alone). In total, 182 participant dyads (patients and their caregivers) will be recruited. On average there will be 3.4 participant dyads per sub-district.

Cluster randomisation is the chosen method of randomisation because first, CBR includes community-level elements and family support groups, based on sub-districts, so individual randomisation is not possible; second, it is logistically more convenient to concentrate CBR workload in fewer sub-districts; and third there is increased acceptability if all participants in a sub-district are allocated to either intervention or control arms. This paper follows the Standard Protocol Items: Recommendations for Interventional Trials (SPIRIT) recommendations for trial protocols (see Additional file 1).

### Setting

The trial will take place in Sodo district, Gurage Zone, Southern Nations, Nationalities and Populations Region (SNNPR) in Ethiopia. The administrative town of Sodo, Bui, lies 100 km from Addis Ababa. Sodo district has a population of 170,000 persons in 58 sub-districts: four urban and 54 rural. Most people live in one-room mud and straw houses and work as subsistence farmers. About 45% of the population are literate and the majority are Orthodox Christian by religion.

There is a psychiatric nurse-led outpatient clinic at the general hospital in Bui. There is a similar unit at Butajira hospital, which is 30 km away from Bui in the neighbouring district. Primary health care is delivered through eight primary health care centres staffed by health officers and general nurses. Each health centre oversees between four and 11 health posts, staffed by two health extension workers (HEWs). Each health post covers a sub-district, with a population of 2800–5000 people. Health care costs are largely out-of-pocket with a free waiver available for the poorest; in practice a tiny minority of the population are likely to be in receipt of this waiver.

### Context of the study

The RISE trial is nested in PRIME (PRogramme for Improving Mental healthcarE). PRIME is a multicountry research consortium that aims to generate evidence on the implementation and scaling up of packages of care for priority mental disorders in primary health care in LMICs [35, 36]. As part of the PRIME project, a comprehensive mental health care plan was developed for Ethiopia [37–39] and, from December 2014, facility-based care for schizophrenia was embedded in primary health care centres in Sodo district. Health officers and nurses were trained in the detection and treatment of schizophrenia, including the prescription of antipsychotic medication and psychoeducation. Training followed the WHO’s mhGAP and evidence-based guidelines [18, 40], adapted for the Ethiopian context. PRIME identified people with schizophrenia in Sodo using the Butajira key informant method [41] and, from December 2014, began following up those invited to access FBC in a 12-month treatment cohort. A subsample of the PRIME cohort participants will be recruited to the RISE trial at the PRIME 6-month data collection. The rationale for recruiting into the trial after participants have had 6 months access to FBC is that if this intervention were scaled up in a resource-poor setting such as Ethiopia, it would only be made available for those with enduring illness or poor treatment outcomes.

### Interventions

#### Facility-based care

FBC will be available to all participants in the RISE trial. The frequency of contact with FBC will be determined by clinical need. Within formal health care, FBC is a three-tier system, but the majority of care will be delivered at tier 1, which is primary care (Table 1).

**Table 1:** Facility-based care three-tier system for the treatment of mental illness

Location	Staff	Tasks	Referral
Tier 1: primary care			
Eight health centres across Sodo district	Health officers and nurses	• Prescribe antipsychotic medication (oral haloperidol or chlorpromazine) and monitor response • Manage side-effects • Monitor suicide risk • Review physical health • Screen for substance misuse • Provide basic psychoeducation (information about schizophrenia) • Provide regular follow-up	• Refer to tier 2 in the following scenarios: o Active suicidal intent o Inadequate oral intake of fluid or food o Risk of violence to others and/or risk of serious self-neglect o Persistently non-adherent to medication o Acutely disturbed and not manageable at home o Pregnancy/breastfeeding o Medical complications o Unmanageable side-effects
Tier 2: general secondary care			
Psychiatric outpatient clinic at Butajira Hospital	Psychiatric nurse	• Specialist review • Initiate depot injection where required	• Refer to tier 3 when inpatient care is required • Refer back to tier 1 for follow-up
Tier 3: specialist care			
Ammanuel Psychiatric Hospital, Addis Ababa	Psychiatrist	• Specialist review • Inpatient admission where required	• Refer back to tier 1 and/or tier 2 for follow-up

### Community-based rehabilitation

#### Intervention development

The CBR intervention was constructed through in-depth intervention development work as described in detail elsewhere [34, 42]. The work included a literature review, situational analysis [43, 44], an intervention development workshop, participatory meetings, qualitative interviews with a range of stakeholders, including people with schizophrenia, their caregivers, psychiatrists and community leaders, and collaboration with an existing CBR project, RAPID (Rehabilitation And Prevention Initiative against Disabilities). RAPID is an Ethiopian CBR project for children with disabilities.

### CBR worker recruitment and training

Eleven CBR workers were recruited according to the criteria: (1) completed tenth grade education (secondary school), (2) resident in Sodo district, and (3) interest in community work. Training lasted for 5 weeks and comprised an equal split of classroom teaching and field work, including home visits to people with schizophrenia and on-the-job training at RAPID. Psychiatrists and CBR trainers from RAPID delivered the training. Competency was assessed using role-plays and patient vignettes.

### Pilot

A 12-month pilot was conducted. The pilot included ten people with schizophrenia and their families living in four sub-districts not involved in the main trial. The aims of the pilot were to determine the acceptability and feasibility of the CBR intervention and to refine the intervention and trial design as needed. Major adjustments to the intervention and trial design were made prior to the trial starting. Minor adjustments may be made to the latter components of the CBR intervention on the basis of pilot findings.

### Trial CBR delivery

One or two CBR workers will be attached to each health centre and each will cover two or three sub-districts. Each CBR worker will have approximately eight people with schizophrenia under their care. The delivery of CBR for schizophrenia will be the only task for the CBR workers. CBR delivery will commence immediately after recruitment into the trial and will continue for 12 months. The CBR visits will take place at the participants’ home and last 30–90 minutes. The intervention is delivered in three phases (Table 2). In phase 1, lasting 2 to 3 months, there are weekly home visits and the focus is on engagement with the family and addressing core needs through compulsory modules such as ‘Understanding Schizophrenia’. In phase 2, lasting approximately 5 to 6 months, home visits are every 2 weeks and address the specific needs of the individual through optional modules such as ‘Getting Back to Work’. In phase 3, lasting approximately 4 months, the emphasis is on preventing relapse as well as maintaining the progress made towards addressing specific needs. CBR workers conduct community mobilisation work and may run family support groups alongside the home visits. The detailed content of CBR at each phase of the intervention is described in Table 1. Flexibility will be encouraged according to the needs of the individual participant and their caregiver. CBR delivery will be guided by a manual that outlines steps for the delivery of each module and community engagement task, procedures for referral and how to deal with difficult situations, for example, suicidal ideation. Two supervisors will maintain an overview of the frequency, content and quality of the home visits by CBR workers for each participant. Supervision will include monthly unannounced observed sessions, with individual face-to-face supervision and group supervision every 2 to 4 weeks. Unannounced visits will take place only with the prior permission of participants. The trial coordinator will attend group supervision monthly and conduct a paper-based review of all cases every 2 weeks with each supervisor. The coordinator will ensure the on-going fidelity of CBR delivery and guide top-up training sessions for CBR workers where required.

**Table 2:** RISE community-based rehabilitation (CBR) intervention outline

Phase	Months	Visits		CBR activities	
			Assessment and family engagement	Community mobilisation	Family-level interventions
1: Intensive engagement	~1–3	Weekly	• Developing therapeutic alliance with family • Needs assessment • Risk assessment • Goal setting for phase 1 • Rehabilitation plan	• Resource mapping for sub-district, e.g. churches, schools, *edir* groups (traditional burial association), Women’s Association, literacy groups, religious groups and traditional healers • Initial awareness-raising and mobilisation, targeting health extension worker, community leaders and traditional healers • Awareness raising meeting/s with general public in sub-district	Core modules: • Understanding schizophrenia • Improving access to health care • Dealing with human rights issues • Preparing for a crisis
2: Stabilisation	~4–8	Fortnightly	• Update needs assessment • Risk assessment • Goal setting for phase 2 • Update rehabilitation plan	• Facilitate access to relevant community resources • Consolidate mobilisation and awareness raising	Optional modules: • Supporting individuals to take medication • Improving the family environment (coping skills, marital problems) • Improving day-to-day functioning • Taking part in community life • Getting back to work • Dealing with distressing symptoms • Dealing with stigma and discrimination • Improving physical health • Dealing with stress and anger • Improving literacy • Family support groups • Taking control of your illness (relapse prevention and management)
3: Maintenance	~9–12	Monthly	• Update needs assessment • Risk assessment • Goal setting for phase 3 • Update rehabilitation plan • Prepare for termination • Continuing care assessment	• Consolidate access to community resources • Consolidate awareness raising and mobilisation	Core module: • Taking control of your illness (relapse prevention and management) if not completed in phase 2Optional modules • Any of the phase 2 modules

### Selection and randomisation of clusters

We will aim to include all 54 sub-districts in Sodo district (after excluding the four pilot sub-districts). The randomisation of sub-districts into CBR plus FBC and FBC arms will be stratified by health centre. A minimisation algorithm [45] will be employed to ensure balance for (1) urban/rural location, (2) number of potential participants (i.e. cases of schizophrenia in the PRIME cohort) in the sub-district, and (3) mean WHODAS 2.0 score in the sub-district at PRIME cohort baseline. All these factors may potentially impact on the primary outcome. PRIME cohort baseline WHODAS 2.0 scores will only provide an estimate of the disability level of potential participants as this data will not be available for the entire pool of trial participants and will not represent disability levels at the time of recruitment. The allocation sequence will be generated randomly from the set of optimal sequences [46]. An independent statistician will generate the allocation list for sub-districts. This list is will be kept securely by the trial coordinator and utilised to determine allocation status of new recruits.

### Participant inclusion criteria

There are no specific exclusion criteria. Participants must meet all of the following criteria to be included: (1) be a participant in the PRIME cohort study or not engaged in FBC but resident in Sodo district, (2) have been resident in the sub-district for more than 6 months and have no immediate plans to leave the sub-district, (3) have a primary caregiver who is willing to participate in the study, (4) be aged 18 years older, (5) have a diagnosis of schizophrenia spectrum disorder (schizophrenia, schizoaffective disorder or schizophreniform disorder) using *Diagnostic and Statistical Manual of Mental Disorders, version four* (DSM-IV) [47] criteria, and (6) have evidence of enduring or disabling illness demonstrated by one or more of the following: (a) Brief Psychiatric Rating Scale – Expanded version (BPRS-E) score ≥52 (equivalent to at least ‘moderately ill’ on the Clinical Global Impression (CGI) scale) [48], (b) 36-item WHODAS 2.0 score ≥35, (c) have continuous illness over the preceding 6 months, as assessed using the Life Chart Schedule (LCS), (4) be symptomatic in 3 out of the last 6 months, as assessed using the  LIFE chart, or (e) have a CGI score ≥3 (at least ‘mildly ill’). The final criterion allows us to include the group expected to benefit the most from CBR and also reflects the threshold at which CBR could realistically be offered in this resource-constrained setting.

### Participant flow

#### Participant identification

PRIME aimed to detect all people with schizophrenia in Sodo by training key informants (HEWs and community leaders) to identify possible cases. This method was successful at identifying people with psychosis in previous Ethiopian studies, including in the neighbouring district [41, 49, 50]. Possible cases of schizophrenia were invited to their health centre for diagnosis and treatment. If they attended, a psychiatric nurse conducted a diagnostic interview using the OPCRIT (Operational Criteria for Research), an operational criteria checklist for psychotic and affective illness [51, 52]. The OPCRIT facilitated the nurse to determine if participants had a DSM-IV diagnosis. Substantial inter-rater reliability and convergent validity of the OPCRIT has been demonstrated in other settings [53] and there is good experience of using OPCRIT in a clinical trial of schizophrenia in the neighbouring district [54]. All confirmed cases of schizophrenia spectrum disorders were offered FBC and, where they consented, were recruited into the PRIME cohort. Prior to RISE recruitment a psychiatrist conducted an additional paper-based diagnosis review of all PRIME cohort participants using the clinical instruments completed at baseline, including the OPCRIT. Where the psychiatrist deemed the diagnosis unclear, a repeat clinical assessment by the psychiatric nurse will be completed prior to RISE recruitment.

### Participant recruitment

Participants for the RISE trial will be primarily recruited from people who were identified and received a diagnosis of a schizophrenia spectrum disorder at the baseline of the PRIME cohort and their caregivers, and were living in the sub-districts selected to participate in the RISE trial. However, we will recruit from up to four pools of potential participants, known as recruitment levels.

*Level 1 recruitment* comprises recruitment from the PRIME cohort at the 6-month data collection interview, which takes place at the health centre. *Level 2 recruitment* comprises recruitment from PRIME cohort drop-outs, i.e. those who do not attend the PRIME 6-month data collection. *Level 3 recruitment* comprises recruitment from those who were identified by key informants but have never attended FBC. This group had some baseline data collected through a home visit by PRIME as part of a non-engagement study. *Level 4 recruitment* comprises recruitment from those individuals who were identified by key informants as potential cases with schizophrenia *after* the recruitment for the PRIME cohort had ended. Level 1 recruitment will take place at consecutive pairs of health centre catchment areas, determined by the PRIME data collection schedule. For levels 2, 3 and 4 recruitment, all sub-districts will be covered equally to avoid unequal recruitment by treatment arm. RISE recruitment will take place either at the health centre at PRIME data collection or at a follow-up visit at the participant’s home.

The aim of incorporating non-PRIME cohort participants is that these non-engagers in care are likely to have more complex needs, and be more symptomatic and disabled. They are, therefore, the individuals who are likely to receive the most benefit from CBR. Some of these individuals will not be accessing care because they are currently well, and will be excluded from the trial using the standard recruitment criteria.

Within each CBR worker area, the transition from one recruitment level to the next will continue until approximately eight participants have been recruited for each CBR worker. The aim is to ensure CBR workers have equal workloads, to ensure CBR delivery is as uniform as possible. If it is not possible to recruit eight participants in any particular CBR worker area (after exhausting all recruitment levels), recruitment will continue at other CBR worker areas whilst aiming to keep the number of participants per CBR worker as equal as possible. Recruitment will proceed to the next recruitment level/s until a maximum of 12 participants per CBR worker are recruited. This is because the maximum feasible workload for CBR workers is expected to be 12 participants. There may be slight adjustments to the number of participants recruited per CBR worker depending on drop-out of CBR workers or other unforeseen factors.

### Recruitment procedures

The recruitment procedures for each of these groups will be detailed in dedicated Standard Operating Procedures (SOPs). Potential participants in the PRIME cohort will have their eligibility for the RISE trial checked by the trial coordinator or trial nurse using PRIME 6-month data (i.e. WHODAS 2.0, BPRS-E, CGI, LIFE chart and LCS). Eligible participants will be invited to join the RISE trial by a trial nurse. They will be given information about the trial in a way appropriate to the participants’ literacy level, which may include verbal information. The trial nurse will then conduct the consent procedures.

Participants not in the PRIME cohort will have an initial consent taken, before data is collected on the eligibility instruments, including the diagnostic interview. Those who are eligible will then be consented to take part in the RISE trial. Those who consent (or, for those without decision-making capacity, whose caregiver consents on their behalf) will complete the full RISE baseline data collection.

### Allocation

The randomisation of sub-districts to intervention and control arms will take place before recruitment. The rationale is that it is necessary to commence delivery of the CBR intervention immediately after recruitment. The trial coordinator will keep the sub-district allocation list secure on a password-protected document. To reduce selection bias, the potential participant will not be informed of the allocation of their sub-district until after they have consented to participate and all baseline data collection is complete. The trial nurse will also be blind to the allocation. The trial coordinator will assign a unique trial identifier (ID) to each new participant and will determine the allocation of all new recruits using the secure allocation list. Recruitment and participant flow will be closely monitored by the trial coordinator and any protocol deviations recorded and reported.

### Outcome assessments

#### Quantitative

All outcomes are individual-level and will be assessed at 6 and 12 months (Table 3). Baseline RISE data will be extracted from 6-month PRIME cohort data and 6-month RISE data will be extracted from 12-month PRIME cohort data. Twelve-month RISE data will be collected independently of the PRIME cohort. Trained lay data collectors will collect all data except for symptom severity, clinical course, overall clinical impression of illness severity and improvement, and medication, for which a trained psychiatric nurse will be used. Patient outcomes will be collected with the caregiver present according to participant preference and when the patient does not have the capacity to answer questions independently. Data will be collected directly from the patient where possible (except for caregiver-reported outcomes). Items will be recorded as missing in the scenario that the caregiver is not present due to patient refusal, and the patient is unable or unwilling to respond. Quality will be assured through systematic observations of data collection by a research assistant (for lay data collectors) or psychiatrist (for psychiatric nurse data collectors), and by verification of all patient record forms. Regular meetings will be held to provide feedback to data collectors.

**Table 3:** Summary of outcome measures

Outcome	Instrument
Psychiatric nurse-administered interview with patient	
Symptom severity	Brief Psychiatric Rating Scale – Expanded version (BPRS- E) [62]. A 24-item instrument focussing on psychotic symptoms, but also covering somatic concerns, anxiety, depression and mania. Individual BPRS-E items and total score are sensitive to change in persons with persistent schizophrenia [63]. The scale has been previously used in Ethiopia [64] and has recently been shown to have good inter-rater reliability (>0.8 comparing psychiatric nurses and psychiatrists) in this setting (personal communication, Dr Charlotte Hanlon) [65]. As the scale is clinician-rated this allows for sociocultural sensitivity. Inter-rater and test-retest reliability as well as internal consistency are also high in high-income settings [66]
Clinical impression	Clinical Global Impression (CGI). A widely used assessment tool, comprising three scales, to determine overall illness severity and efficacy of intervention [67]
Relapse	Life Chart Schedule (LCS) including course type and relapses [68].
	Longitudinal Interval Follow-up Evaluation: DSM-IV version (LIFE). A semi-structured interview to determine the subject’s psychiatric course since the last interview [69]. Satisfactory validation has been conducted in Ethiopia and the reliability data is currently being analysed. Any necessary adjustments will be made on the basis of the inter-rater reliability assessments (personal communication, Dr Girmay Medhin)
Lay data collector-administered interview with the patient	
Disability	Patient-reported 36-item WHODAS (Disability Assessment Schedule) 2.0 [55].
	A validated indigenous functioning scale, specific to the Ethiopian context [70]
Economic activity	Measure covering current employment, subsistence farming work, income, and hunger due to lack of resources
Discrimination	Section 1 of the Discrimination and Stigma Scale-12 (DISC-12) [71]
Medication adherence	Adapted Morisky Medication Adherence Scale (MMAS) [72]. This scale has previously been utilised for antipsychotic medication adherence [31, 73]. Good construct validity has been demonstrated in Ethiopia (personal communication, Dr Charlotte Hanlon) [65]).
	A 5-point nominal scale measuring frequency of adherence [31]
Health service use and costs including engagement with FBC for schizophrenia and physical health conditions	An adapted version of The Client Service Receipt Inventory (CSRI) will enquire systematically about the costs (direct and indirect) of help-seeking from biomedical, traditional and religious healers [74, 75]. The CSRI has been translated into Amharic and found to be acceptable and feasible [76]
Access to community interventions (including CBR components)	Including person administering the component, and satisfaction
Physical restraint	In the preceding 1 and 6 months. Includes assessment of duration, perpetrator, setting and reason for restraint
Nutritional status	Measurement of weight (kg) and height (m) will be carried out [77] and body mass index (weight in kg/(height in m)^2^) calculated
Depression	The Patient Health Questionnaire- 9 (PHQ-9). A 9-item scale which scores each of the 9 DSM-IV criteria for depressive disorders as ‘0’ (not at all) to ‘3’ (nearly every day) [78]. Shown to be valid in the Ethiopian setting [79]
Alcohol use disorder	The AUDIT (Alcohol Use Disorders Identification Test) is a ten-item tool to detect hazardous drinking [80]
Social support	Oslo-3 Social Support Scale [81]
Serious adverse events	Occurrence of serious adverse events (for example, suicide attempt and hospitalisation for medical emergency) in the last 6 months
Lay data collector interview with the primary caregiver	
Patient disability	The 36-item WHODAS 2.0 proxy version will assess functional impairment from the caregiver’s perspective [82, 83]
Economic activity of caregiver	Employment, subsistence farming work, income, and hunger due to lack of resources
Caregiver burden	The Burden Section of the WHO ‘Family Interview Schedule’. This scale, covering financial strain and work difficulties has been previously used in Ethiopia for persons with schizophrenia [2].
	A scale developed for PRIME will quantify the time burden of caring for their relative with schizophrenia, the type of work that was stopped or reduced and the amount of money lost. The Involvement Engagement Questionnaire (IEQ) will be used as an additional measure to assess caregiver burden.
Caregiver depression	PHQ-9 [78, 79]
Stigma	Section of the WHO ‘Family Interview Schedule’; previously used in Ethiopia [8]
Patient medication adherence	The 5-point nominal scale developed for the COPSI study will be used [31]

*CBR* community-based rehabilitation, *DSM-IV Diagnostic and Statistical Manual of Mental disorders, version four*, *FBC* facility-based care

In order to reduce information bias data collectors will be masked to sub-district allocation; participants will be requested not to divulge treatment allocation to data collectors; participants from different sub-districts will be allocated to each data collector; identical methods for follow-up will be employed in each arm; and those involved in data analysis will be masked to sub-district allocation.

Loss to follow-up will be minimised by reminding participants to attend the interview shortly before they are due, either by telephone or by a home visit. Participants who do not attend will receive a home visit to ask them to attend at an alternative time. Data collection will take place at the health centre except for participants who do not attend after three invitations. These participants will receive a home visit for data collection. Participants will receive a modest fee for transport and time compensation.

The primary outcome is proxy report of patient disability which will be measured with the 36-item WHO DAS (Disability Assessment Schedule) 2.0 [55], through interviewing the caregiver. WHODAS 2.0 was developed as single generic instrument for assessing health status and disability relating to a range of health conditions across cultures and settings. It covers understanding and communication, getting around, self-care, getting along with people, life activities and participation in society. Sociocultural adaptation and validation (convergent validity, construct validity and responsiveness to change) of the WHODAS 2.0 in persons with schizophrenia has been completed in Ethiopia (personal communication, Kassahun Habtamu). Issues arising during the adaptation included items not having obvious direct translations and representing uncommon experiences in the rural Ethiopian setting (for example, ‘staying by yourself for a few days’). These issues were largely resolved through iterative adjustments to the translation following piloting. The proxy WHODAS 2.0 interview is designed to be answered by a friend, relative or carer. The rationale for using the proxy version for the primary outcome is that this may give a more valid picture of disability level. A separate study in the neighbouring district found that whilst scores from patient- and proxy-reported versions are moderately correlated, there is a difference, with the caregiver scores tending to be higher (greater disability) (personal communication, Kassahun Habtamu). Patients may not have enough insight to answer accurately and, therefore, underreport disability. They may also be too unwell to answer all questions, increasing the amount of missing data. Table 3 lists the secondary outcomes, which include patient-reported WHODAS 2.0, symptom severity, relapse, medication adherence, economic activity in the patient and caregiver, discrimination, health service use and costs, physical restraint, nutritional status, caregiver burden, caregiver depression and caregiver stigma. All instruments have been adapted for use in Ethiopia; further details on validation and reliability are also given in Table 3.

### Process data

Process data will be compiled using a range of sources to determine the quality and intensity of intervention delivery. These include (1) CBR worker ‘sub-district’ logbooks, including type and number of community engagement tasks completed, (2) CBR worker ‘participant’ logbooks including home visit forms (modules undertaken, duration and travel time) and assessment forms, (3) monthly observation of CBR visits by supervisor and completion of the ENACT (Enhancing Assessment of Common Therapeutic factors) rating scale [56] adapted and validated for the Ethiopian context. The adapted ENACT assesses communication skills, engagement with the individual, family and community, assessment of medication adherence, physical health, substance use and suicide risk assessment, (4) observation of CBR home visits by an external clinician (psychiatric nurse or psychiatrist) to complete the ENACT, providing an independent assessment of CBR worker skills, (5) CBR worker competency assessment by supervisor (rating of 1 to 3 given for each of 47 CBR competencies, e.g. ability to conduct community awareness raising meeting), (6) CBR worker self-assessed competency form, (7) supervisor logbook including number of supervision sessions attended, (8) health centre records to determine frequency of contact with participant, and (9) participant-reported structured assessment of the extent to which CBR met their needs.

### Qualitative

In-depth interviews (IDIs) will be conducted at baseline and 12 months with a sub-sample of patients and caregivers to gather information on the impact of CBR and other factors on the experience of illness and recovery. Around eight to ten participant dyads from each treatment arm will be included depending on when theoretical saturation is reached. After gaining separate informed consent, interviews will be audio-taped, transcribed in Amharic and then translated into English prior to conducting a thematic analysis. IDIs will be conducted with four to eight community leaders to understand their role in CBR and their perception of its potential impact. IDIs and FGDs will be conducted with CBR workers and supervisors to understand their experience of delivering CBR and its perceived impact on participants. One or two focus groups will also be held with health centre nurses to understand their experience of working with CBR workers.

### Power calculation

We estimate that 182 participant dyads (mean 3.4 participants/sub-district) will be available for recruitment. Assuming that there is 23 % attrition, the final sample size for analysis will be 140 participant dyads in 54 sub-districts (mean 2.6 participants/sub-district). This sample size will allow us to detect a 20 % absolute difference in WHODAS 2.0 scores between treatment arms with 85 % power and 5 % significance, assuming a *k* (coefficient of variation) of 0.14 and a within-cluster standard deviation (SD) of 16. The value of *k* was extracted from symptom severity data by sub-district from an RCT in Butajira evaluating trimethoprim as an adjuvant treatment for schizophrenia (*k* = 0.11) [57]. A more conservative estimate of *k* has been used to account for the potential therapist effect; each CBR worker will cover around three sub-districts and CBR intensity and quality may differ by CBR worker. The mean WHODAS 2.0 in intervention and control arms and within-cluster SD were derived from an Indian study of people with schizophrenia [58].

The estimate of 182 participant dyads available for recruitment was arrived at using (1) the total adult population of Sodo district, (2) the local prevalence of schizophrenia (0.05 %) [59], (3) an estimate that 60 % of cases will be detected and agree to participate in the PRIME cohort, (4) an assumption that 70 % of cases will be eligible for RISE, based rates of continuous illness found in a previous study in the neighbouring district of Butajira [5], and (5) an assumption that approximately 80 % of eligible cases will agree to participate, based on previous recruitment rates in the Butajira cohort study [5].

### Data management

All data collection and management will follow Good Clinical Practice (GCP) guidelines and SOPs. Each participant has a unique ID that will enable all data reported for each participant to be identified. Blinding will be maintained during data collection, entry, processing and primary data analysis. Data is collected on paper Patient Report Forms (PRF) and double entered onto electronic Case Report Forms on EpiData Entry Client (2.0.7.22). Data will be managed using EpiData Data Manager (2.0.8.56).

All databases will be password-protected and only accessible to authorised personnel. Data cleaning based on frequency distributions and logic checks will follow standard procedures with reference to source documents as required. The data system is designed to ensure that all data changes are documented and that there is no undocumented deletion of entered data, i.e. an audit trail will be in place. Systematic checks will be carried out to ensure that the audit trail is functioning correctly. Data and all appropriate documentation will be stored for a minimum of 5 years after the completion of the study, including the follow-up period.

### Data analysis

Data analysis will take place using Stata, version 13. The primary outcome analysis will be masked until the analysis is finalised and approved by all investigators. Adequate CBR will be defined as having received a minimum of ten home visits by a CBR worker. We will not define adequate treatment for the FBC arm a priori. Sociodemographic and clinical characteristics (including WHODAS 2.0 score) of eligible PRIME cohort participants who did and did not consent to RISE will be compared using chi-square tests and *t* tests. Loss-to-follow-up will be compared by treatment arm at 6 and 12 months to assess bias due to loss to follow-up. Of those who enrolled, descriptive summaries of sociodemographic and clinical characteristics, presented by treatment arm, will be produced for baseline data. CBR worker characteristics such as age, gender, education level and post-training competency will be described.

Endpoint data will be analysed under intention-to-treat assumptions. An individual-level analysis will be conducted using a multilevel, mixed-effects regression model to compare the WHODAS 2.0 score between treatment arms, accounting for clustering at sub-district and CBR worker levels. The WHODAS 2.0 distribution will determine the model used. If zero-inflation is detected, a zero-inflated negative binomial model will be considered [60]. Adjustment will be made for baseline WHODAS 2.0 score and covariates unbalanced at baseline. Logistic regression will be used to assess which baseline variables are associated with missing outcome data. Baseline variables that predict missing outcome will be included in the regression models as fixed covariates to meet the assumption that outcome data is missing at random. Multiple imputation methods will then be used for participants with missing outcome data.

Sensitivity analyses will include a complete case analysis and a complier average causal effect (CACE) analysis [61]. We will also assess whether there is a dose-response relationship between degree of adherence to CBR (i.e. number of sessions) and the primary outcome. A further analysis will be undertaken to understand which the active components of CBR are. Process data on which CBR components were received, and in what quantity, will be utilised. Potential correlations between different components will be taken into account.

Exploratory sub-group analyses by baseline symptom severity and antipsychotic medication adherence will be completed, although the study will not be powered to investigate such differential effects. A longitudinal analysis will be conducted using a random-effects model. Three levels (CBR worker, sub-district and individual) and two time points (6 and 12 months) will be included. Secondary and tertiary outcomes will be analysed using mixed-effects linear regression models or logistic regression depending on the data type.

### Cost-effectiveness analysis

Direct costs of the treatment will be estimated by deriving a monetary value for each component of the treatment based on actual costs, and applying these to each individual based on the process indicators, which reflect the actual uptake of the treatment. Other health care costs and other patient- or family-borne costs will be computed and compared at 6 and 12 months, and subsequently related to changes in disability and clinical symptoms. Incremental cost-effectiveness ratios will be calculated to illustrate the extra cost incurred (if any) to produce a unit improvement in the main outcome of disability-adjusted life years (DALYs) calculated from the 36-item WHODAS 2.0. In the event that dominance is not shown, i.e. the intervention is more effective but the costs are also more than the FBC group, incremental cost-effectiveness ratios will be computed, together with their confidence intervals (using bootstrapping techniques to overcome expected skewness of cost data). Cost-effectiveness acceptability curves will also be derived in order to show the probability of any cost-effective advantages for the FBC plus CBR group at a range of ‘willingness to pay’ threshold levels.

The results from the quantitative and qualitative work will be combined to give an overall assessment of the intervention effectiveness.

### Trial management and monitoring

The trial is sponsored by the London School of Hygiene and Tropical Medicine. A Data Safety and Monitoring Board (DSMB) has been convened to assure the continuing safety of research participants. A clinical monitor will carry out onsite monitoring visits prior to the trial commencing, at recruitment, and at 6-month and 12-month follow-up. The trial coordinator will carry out day-to-day monitoring and will also review recruitment rates, withdrawals and losses to follow-up. All protocol violations will be recorded and included in reports of trial findings.

### Confidentiality

Trial-related assessments will take place in private locations. A unique ID number will be linked to patient details in hard and soft copy formats that are kept in secure locations. Signed consent forms will be kept securely in a locked cupboard. All documentation that includes patient data will be anonymised, but identifiable through the ID number. Names of patients will not be quoted or published.

## Discussion

Human rights violations and the high disability burden of schizophrenia make the condition a priority area for public health action. The importance of rehabilitation services for schizophrenia in scaling up mental health care is recognised by the WHO [18] and the Ethiopian government [32]. There is emerging evidence that CBR for schizophrenia is effective at improving disability outcomes in middle-income settings, yet there is no evidence on whether CBR can work in a setting with even fewer mental health resources, such as Ethiopia. The RISE trial will determine the added value of CBR compared to FBC alone. This will help to determine, for scaling-up services, the importance of a dedicated rehabilitation service in addition to FBC.

## Trial status

Recruitment complete; trial ongoing.

## Abbreviations

BPRS-E, Brief Psychiatric Rating Scale – Expanded version; CBR, Community-based rehabilitation; CGI, Clinical Global Impression; COPSI, Community care for people with Schizophrenia in India; DSM-IV, *Diagnostic and Statistical Manual of Mental Disorders, version four*; ENACT, ENhancing Assessment of Common Therapeutic Factors; FBC, Facility-based care; FGD, Focus group discussion; HEW, Health extension worker; IDI, In-depth interview; IRB, Institutional Review Board; LCS, Life Chart Schedule; LMICs, Low- and middle-income countries; LSHTM, London School of Hygiene and Tropical Medicine; mhGAP, Mental health Gap Action Programme; PRIME, PRogramme for Improving Mental health carE; RCT, Randomised controlled trial; RISE, Rehabilitation Intervention for people with Schizophrenia in Ethiopia; SOP, Standard Operating Procedure; WHO, World Health Organisation; WHODAS 2.0, WHO Disability Assessment Schedule, version 2.0

## Additional file

Additional file 1: SPIRIT checklist – RISE trial protocol. A checklist of the full protocol components according to the SPIRIT criteria. (PDF 106 kb)
